# Shoot Feeding as a Nutrient Acquisition Strategy in Free-Living Psylloids

**DOI:** 10.1371/journal.pone.0077990

**Published:** 2013-10-23

**Authors:** Martin J. Steinbauer

**Affiliations:** Department of Zoology, La Trobe University, Melbourne, Victoria, Australia; Federal University of Viçosa, Brazil

## Abstract

Shoot feeding by sucking insects is accepted as an adaptation to feeding where plant nutrients are most concentrated and/or of higher quality. Psylloids are an important hemipteran taxon, most of which are free-living and comprise many shoot feeding species, whose nutritional ecology has been largely ignored. I conducted a longitudinal study of *Ctenarytaina eucalypti* (Maskell) and *C. bipartita* Burckhardt et al. (Aphalaridae) feeding on eucalypts to document how within-plant (ontogenic) variation in nutritional quality, in particular of free amino acids, determines host suitability and hence the distribution and abundance of nymphs. Nymphs were most abundant within developing apical buds but were not more abundant on branchlets of greater vigour (indicated by rate of extension). Nymphs could be found up to two (*C. bipartita*) to three (*C. eucalypti*) alternate leaf pairs distant from apical buds but infrequently and in low numbers; they were never found on older, fully expanded leaves. The position of a leaf on a branchlet (indicative of age) determined its nutritional quality. Younger leaves had higher water contents, lower chlorophyll contents and differed in amino acid (essential and non-essential) composition compared to older leaves. The abundance of *C. eucalypti* nymphs on expanding leaves and in buds was positively correlated with the concentrations of methionine, valine and threonine in *E. globulus* leaves at the same or comparable position on a branchlet. The abundance of *C. bipartita* nymphs was positively correlated with foliar leucine concentrations. Shoot feeding by these two psyllids facilitates access to more concentrated, better quality plant nutrients but may not entirely explain the adaptive significance of their behaviour. The humid microclimate created by the architecture of the hosts’ apical buds protects eggs and nymphs from desiccation and is suggested to have had a significant influence on the evolution of host utilisation strategies of psyllids within this genus.

## Introduction

 Shoot feeding by sucking insects is generally accepted as an adaptation for the utilisation of the highest quality modules available on a host, often at a specific time. In shoot feeding aphids, peaks in their abundance coincide with flushes in host plant growth [1,2]. Populations of these “flush feeders” exhibit their greatest abundance on high quality plant modules [3,4]. In many instances, the highest quality plant modules are shoots exhibiting vigorous vegetative growth [5,6]. Aphid performance has been shown to be related to changes in phloem amino acid composition associated with the host’s developmental stage. Specifically, aphid feeding declined on hosts directing resources into tuber maturation which is a time when the phloem concentration of glutamine decreases [7]. This nutritionally driven change in behaviour and its consequence for aphid reproductive performance ultimately has population-level significance, e.g. populations crash when host quality becomes unsuitable in mid-summer [8]. Responses such as these are recurring themes in aphid population ecology [e.g. 9,10,11] and seem to have direct parallels in psylloids. For example, Sutton [12] reported that individuals of two species of psyllid were larger when feeding on flowering *versus* non-flowering hawthorn and suggested that size differences were related to the higher levels of soluble nitrogen in the tissues of the former. Temporal variation in the nutritional quality of eucalypts has also been implicated in the onset of seasonal dormancy (“oligopause”) in *Ctenarytaina eucalypti* [13].

 Psylloids, or psyllids (Hemiptera: Sternorrhyncha), are entirely phytophagous sucking insects found throughout the major zoogeographical regions of the world. Worldwide, the superfamily Psylloidea comprises approximately 3,000 to 3,500 described species in some 270 genera [14,15]. In common with aphids, the economic significance of certain exotic species of psyllid relates primarily to their capacity to vector harmful plant pathogens [16,17,18]. To-date, no Australian psyllid species has been reported to vector a plant pathogen harmful to their eucalypt host. The main detrimental effects of psyllids on eucalypts in Australia include the ability of some (notably *Cardiaspina* species) to case leaf chlorosis, necrosis and premature abscission [19,20] and of others (e.g. *Glycaspis* species) to support the growth of smothering sooty mould on leaf surfaces [20]. Psyllid nymphs develop via one of four distinct biologies, namely free-living, gall-forming, lerp-forming or as inquilines. In Australia, some 40% of species are free-living, 50% are lerp-forming and 10% are gall-forming [21]. Lerps are scale-like coverings under which the nymphs develop; the behaviour is exhibited by Australian species of Spondyliaspidinae, Eastern Palaearctic *Celtisaspis* species (Pachypsyllinae), Neotropical *Euphalerus* species (Euphalerinae) and Afrotropical *Retroacizzia* species (Euphalerinae)) [22]. Nymphal biology presumably has significant implications for diet composition. Gall formation has been shown to re-direct amino acids to the insects that induce them [23,24] while feeding by lerp-forming *Cardiaspina* psyllids has been suggested to induce premature leaf senescence and the mobilisation of nutrients thereby making them available to developing nymphs [4,25]. Given that the free-living biology is probably plesiomorphic, the feeding strategy of these species is likely the simplest, involving development where plant nutrients are being directed for growth. That this might be the nutrient acquisition strategy of shoot feeding psyllids was suggested by [26,27].

 Unlike our understanding of the importance of amino acids in aphid nutritional ecology (as well as some whiteflies and leafhoppers), little has been published on their influence on psyllids. Of the three studies known to the author, one did not quantify concentrations of individual amino acids [28] and another does not relate the concentrations of individual amino acids to psyllid survival or development [29]. The third study found that concentrations of isoleucine decreased when heather was grown under increased UVB and it was suggested that this might explain the decline in abundance of the psyllid *Strophingia ericae* [30].

 To understand psyllid-plant interactions and their consequences for psyllid population dynamics, it is essential that psyllid nutritional ecology be understood in greater detail. In particular, the influence of the physiological status of the host on amino acid composition and its capacity to affect psyllid survival requires more attention given the findings of previous research [see 31]. Since more than two-thirds of Australian psyllid species are host specific for *Eucalyptus* trees, we need to supplement studies conducted under controlled conditions with studies conducted in forest ecosystems; these two situations necessitate adoption of different experimental techniques. Consequently, I conducted a longitudinal study of two free-living psyllids to test the hypotheses that shoot feeding is (1) associated with the utilisation of more vigorous plant modules and (2) provides access to more concentrated plant nutrients as opposed to feeding on expanded (older) plant modules. Answers to these hypotheses should help partition causal explanations of the distribution and abundance of psyllids between and within individual hosts. If host nutritional quality cannot entirely explain psyllid occurrence, the importance of other factors may need to be considered, e.g. behavioural cues on site-specific oviposition [32].

## Materials and Methods

### Focal Psyllids

 The genus *Ctenarytaina* comprises 15 described species endemic to the Austro-Oriental and Pacific region as well as another three currently misplaced species; it is one of the largest psyllid genera found on *Eucalyptus*. The blue gum psyllid, *C. eucalypti*, is so-called because it is commonly found on Tasmanian blue gum, *Eucalyptus globulus* Labill., producing juvenile foliage. The other psyllid studied, *C. bipartita*, is found naturally on juvenile *E. kitsoniana* Maiden and *E. viminalis* Labill [33].. The nymphs of both species secrete waxy filaments that partially cover them unless dislodged by wind. Neither insect nor tree species is listed in Australia as endangered or protected.

### Study Sites and Psyllid Surveys

*E. globulus* subspecies *bicostata* (Maiden, Blakely & J.Simm.) Kirkpatr. or *pseudoglobulus* (Naudin ex Maiden) Kirkpatr. growing at a location near Waterford Park, Victoria (145°3’11.36”E, 37°17’22.90”S; 271 m a.s.l.), were chosen for studying *C. eucalypti* and *E. kitsoniana* growing on Hoddle Range, Victoria (146°7’55.60”E, 38°43’0.90”S; 254 m a.s.l.), were chosen for studying *C. bipartita*. Both locations are on privately owned land; permission to access each location was granted by Tim McDonnell (Waterford Park) or Andrew and Lyn Jamieson (Hoddle Range). The canopies of *E. globulus* comprised 95-100% juvenile foliage (range in tree height in October 1.4 to 2.9 m) whereas the canopies of *E. kitsoniana* comprised only 5-10% juvenile foliage (range in tree height in October 2.2 to 4.3 m). I followed psyllids on 28 infested branchlets on seven trees of each species over a period of five months from October 2011 (second month of the austral spring) until February 2012 (last month of the austral summer). To provide sufficient time for appreciable branchlet extension and associated expansion of new leaves, psyllid surveys and leaf measurements occurred at monthly intervals. At each survey, the number of nymphs in the apical bud and on each alternate leaf was recorded.

### Psyllid Fecundity

 Branchlet length and chlorophyll index measurements initially suggested that *E. kitsoniana* may be the less vigorous and nutritious host (latter inference based on relationships between SPAD values and foliar nitrogen given in [34]). To determine whether differences in psyllid abundance might be due to differences in fecundity or host quality, the number of offspring produced by each species of psyllid was examined. Twelve pairs of adults of each species from glasshouse colonies derived from the same source populations as studied in the field were confined on individual seedlings of their host by means of 24 cm long lengths of 7 cm diameter polycarbonate tubing sealed at one end with 0.5 × 0.5 mm mesh; each tube had two 14 mm mesh covered ventilation holes equidistant along its length. Seedlings were grown in 12.5 cm diameter plastic pots containing a commercial Australian native plant potting mix. Seedlings supporting psyllids were grown in an Adaptis CMP 6010 (Conviron, Winnipeg, USA) cabinet with a 20/10 °C for 14:10 h temperature regime; the temperature regime matched the light:dark regime. Each seedling was given 200 mL of water every second day. All nymphs and adults on each seedling were counted after 24 and 27 days, respectively.

### Branchlet and Leaf Measurements

 Branchlets were tagged using small, coloured cableties. To track branchlet extension and leaf production, I numbered leaf pairs in sequence from oldest to youngest relative to a biologically meaningful reference point. On each branchlet, I located what I called the “scale leaves” to identify the location where shoot extension had resumed. This small pair of leaves was assumed to be either the last pair expanded before shoot extension ceased prior to winter (eucalypt leaves become smaller and thicker in response to decreasing temperature and photoperiod [35,36]) or the leaves Ashton [37] likened to cataphylls surrounding the main bud that are abscised shortly after shoot extension resumes. The scale leaf pair was numbered zero with older leaves numbered negatively and younger leaves numbered positively; leaves with the same numbering on different branches of the same tree or on different trees were considered to be the same relative age. Branchlet length was measured from the node where the scale leaves arose to the node at the base of the bud; the change in length between consecutive surveys was used as an indicator of host branchlet vigour.

 Rather than quantify leaf traits for all leaves on a branchlet, alternate odd numbered leaf pairs were chosen for study. Leaf water content and chlorophyll index measurements were taken as indicators of host quality. One leaf of each pair was harvested for estimation of water content. Leaves from tagged branchlets were kept in individual zip-lock plastic bags on ice blocks <-4 °C after harvesting. Upon their return to the laboratory, the fresh weight of each leaf was recorded before being placed in an individual envelope and oven-dried at 70 °C for 24 h after which time they were re-weighed; gravimetric water content was calculated using the difference in weights. A Minolta SPAD-502 meter was used to take chlorophyll index measurements from the abaxial surface of the remaining leaf; measurements from the same approximate location were taken at each survey. SPAD measurements could not be taken on bud leaves since they did not provide a flat surface for the measuring head of the meter. Bud leaves were also not harvested for water content or amino acid analysis because removal of a leaf would have increased nymphal mortality and one bud leaf would not provide sufficient material for LC-MS analysis of free amino acids.

### Amino Acid (AA) Analysis

 Since this study sought to document how within-plant (ontogenic) variation determines host suitability for these two psyllids, the nutritional quality (i.e. water + AAs) of individual leaves on tagged branchlets needed to be quantified. Consequently, I harvested whole leaves of each species on three occasions for analysis of free AAs. *E. globulus* leaves were harvested on 26 October and 7 December 2011 and 8 February 2012 while *E. kitsoniana* leaves were harvested on 2 November, 14 December and 15 February. The October and November harvests of *E. globulus* and *E. kitsoniana* leaves, respectively, could not be coincident with a psyllid survey as were the other two harvests; for logistical reasons each of these harvests had to occur a fortnight after the psyllid survey. For the first two harvests, one branchlet per tree with the same number of leaf pairs as the tagged branchlet with the highest number of leaf pairs was located and leaves at corresponding leaf pair positions harvested. The midrib was cut from each leaf after harvesting before they were placed in individual envelopes inside zip-lock plastic bags and kept on dry ice -78 °C. Nitrile examination gloves were worn during leaf harvesting and later handling. Immediately on their return to the laboratory, leaf halves were microwaved for 30 s in a 1150 W conventional microwave oven, before being oven-dried at 70 °C for 24 h (adapted from [38]). Dried leaf samples were stored at -80 °C prior to analysis.

 Amine group containing metabolites were quantified following the technique described in [39]. Briefly, 30 mg of homogenised leaf was weighed into 2 mL cryo-mill tubes (Sapphire Biosciences, Waterloo, NSW, Australia) to which was added 250 μL 50% aqueous-MeOH solution containing the internal standard ^13^C_5_^15^N-valine (50 µM), which was followed by vortexing. Samples were homogenized (three × 45 s at 6,100 rpm with 45 s rest) using a cryo-mill (Bertin Technologies, France) followed by incubation for 15 min at 70 °C in a thermomixer at 850 rpm. Samples were centrifuged for 15 min at 1,300 rpm after which the supernatant was transferred to 2 mL Eppendorf tubes. 200 μL of MQ water containing 1% formic acid was added to the pellet remaining in each cryo-mill tube which was then vortexed and centrifuged again for 10 min at 1,300 rpm. The supernatant was transferred to the 2 mL Eppendorf tube containing the corresponding MeOH supernatant. This process was repeated before the solution was vortexed, 200 μL of chloroform added followed by vortexing. Solutions were centrifuged for 5 min to form a biphasic mixture consisting of approximately 450 μL aqueous extract and 400 μL organic extract. A 10 μL aliquot of the top polar phase was taken for derivatisation with 6-aminoquinolyl‑*N*‑hydroxysuccinimidyl carbamate (Aqc) followed by LC-MS analysis.

 Data for 19 of the 20 common AAs and three other amine group containing metabolites (latter comprising 4-hydroxy-proline, ornithine and γ-aminobutyric acid (GABA); following [40]) are presented. The amino acids designated herein as essential are those identified by Douglas [41]. Responses for histidine were poor and often below limits of detection or quantitation, hence data for this AA could not be presented.

### Statistical Analyses

 Counts of nymphs associated with leaf pairs and buds of tagged branchlets were square root (count + 0.5) transformed prior to statistical analysis. Monthly abundances of nymphs were compared using ANOVA with Tree, Branchlet nested within Tree and Leaf pair as factors; Tree and Branchlet were treated as random factors while Leaf pair was treated as a fixed factor. These analyses were performed using MINITAB 13.32. Branchlet length over four time intervals (Oct-Nov, Nov-Dec 2011, Dec-Jan 2012 and Jan-Feb 2012) was analysed by repeated-measures ANOVA followed by Tukey B *post-hoc* tests; these analyses were conducted using SPSS 18.0.0. The decline in branchlet extension over the course of the study determined the number of new leaves for which SPAD and leaf water content measurements were taken, in particular during January and February. This influenced the statistical models and comparisons able to be made using these data. Percentage leaf water content was arcsine transformed prior to ANOVA with Month (*E. globulus* only), Tree nested within Month, Branchlet nested within Tree and Leaf pair as factors; Month, Tree and Branchlet were treated as random factors while Leaf pair was treated as a fixed factor. These analyses were also performed using MINITAB. SPAD measurements from leaves extant or expanded during the same survey were compared across consecutive months to reveal changes in chlorophyll concentration. SPAD measurements were also analysed by repeated-measures ANOVA using SPSS 18.0.0. Concentrations (in pico moles mg^-1^) of free AAs were log_10_ (concentration + 1) transformed prior to statistical analysis. Multivariate abundance analysis with R version 2.15.1 [42] was used to examine how concentrations varied according to Leaf pair and Month. Univariate tests were used to identify individual amino acids with the greatest influence on the multivariate results. Pearson correlation matrices were used to identify relationships between the abundances of nymphs in buds and branchlet length, leaf water content (of the youngest leaf) and SPAD measurement (of the youngest leaf). Since the concentrations of many AAs are intercorrelated, factor analyses using the principal extraction technique based on covariance matrices and varimax rotation were performed before considering relationships between nymphs and concentrations of AAs. Stepwise regression using component factor scores was then used to consider relationships between the abundances of nymphs and groupings of AAs. These analyses were conducted using SPSS 18.0.0. Raw data graphing was used to examine how nymphal abundances were related to concentrations of essential AAs identified by stepwise regression as having explanatory significance. AA data for the three leaf pairs closest to the bud were graphed. AA data for the February harvest are not presented since they could not be related to the abundance of psyllid nymphs.

## Results

### Psyllid Abundance

**Figure 1:**
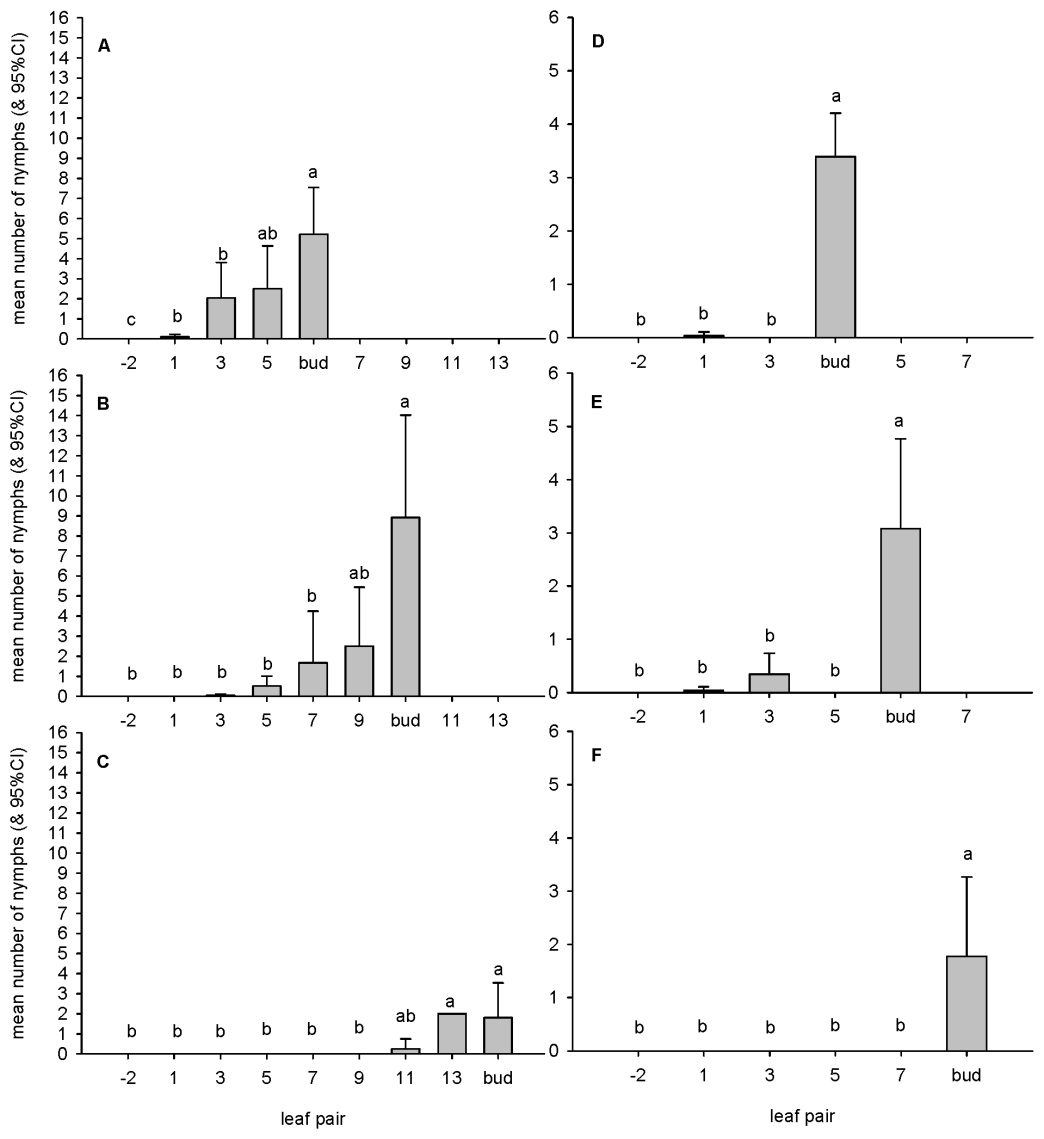
Distribution and abundance of psyllid nymphs. **A**, **B**, **C**. *C. eucalypti* on *E. globulus* in October, November and December 2011, respectively. **D**, **E**, **F**. *C. bipartita* on *E. kitsoniana* in October, November and December 2011, respectively. Similarities of means, determined by *post-hoc* tests, indicated by superscripted letters above bars in individual figures. Note different scale on y-axes for each species of psyllid and the position of the bud at each survey.

 Nymphs of both species were not found on tagged branchlets after the December survey. *C. eucalypti* nymphs were more abundant than *C. bipartita* nymphs (Figure 1). Nymphs of *C. eucalypti* were most abundant in buds but could be found up to three leaf pairs distant from the bud (Figures 1a-c). This close association is evident in the highly significant result for Leaf pair shown in Table 1. The statistically significant result for Branchlet in October was due to a single count of 32 nymphs in the bud of a tagged branchlet, i.e. double the next highest count recorded in the same month. Else wise, abundances of *C. eucalypti* nymphs did not differ according to either Tree or Branchlet. The association of *C. bipartita* nymphs with the buds of host branchlets was more pronounced than that displayed by *C. eucalypti*; nymphs of *C. bipartita* were rarely found up to two leaf pairs distant from the bud (Figures 1d-f; Table 1).

**Table 1:** Analyses of psyllid nymph abundance.

	Source	d.f.	*F*-ratio	*P*
*C. eucalypti*				
Leaf pairs -2 to 5 & bud; Oct	Tree	6	1.40	0.262^*^
	Branchlet (Tree)	21	1.91	0.020
	Leaf pair	4	22.53	< 0.001
	Error	86		
Leaf pairs -2 to 9 & bud; Nov	Tree	6	0.58	0.745^*^
	Branchlet (Tree)	21	1.34	0.162
	Leaf pair	6	12.95	< 0.001
	Error	115		
Leaf pairs -2 to 13 & bud; Dec	Tree	6	1.08	0.402^*^
	Branchlet (Tree)	21	0.98	0.496
	Leaf pair	8	7.15	< 0.001
	Error	141		
*C. bipartita*				
Leaf pairs -2 to 3 & bud; Oct	Tree	6	0.75	0.618^*^
	Branchlet (Tree)	21	1.20	0.277
	Leaf pair	3	79.11	< 0.001
	Error	65		
Leaf pairs -2 to 5 & bud; Nov	Tree	6	0.53	0.778^*^
	Branchlet (Tree)	21	1.30	0.202
	Leaf pair	4	17.56	< 0.001
	Error	78		
Leaf pairs -2 to 7 & bud; Dec	Tree	6	1.01	0.441^*^
	Branchlet (Tree)	20	0.68	0.827
	Leaf pair	5	9.98	< 0.001
	Error	66		

Abundances analysed using ANOVA.

x not an exact *F*-test

### Psyllid Fecundity

 After 27 days, the average increase in the number of *C. eucalypti* on each potted host was 41.5 psyllids (*n* = 11; one pair of adults failed to oviposit). This represents an average rate of increase of 1.54 ± 0.25 (± SE) offspring per day. By contrast, after 24 days, the average increase in the number of *C. bipartita* on each potted host was 60.7 psyllids (*n* = 11; one pair of adults failed to oviposit). This represents an average rate of increase of 2.53 ± 0.34 offspring per day. A *t*-test of the difference between these two rates of increase was statistically significant (*P* = 0.014). *C. bipartita* nymphs developed faster than *C. eucalypti* nymphs. On six potted hosts, between 8.2 and 26.7% of nymphs had reached adulthood, whereas, no *C. eucalypti* nymphs had reached adulthood despite being left to develop for longer.

### Branchlet and Leaf Development

**Figure 2:**
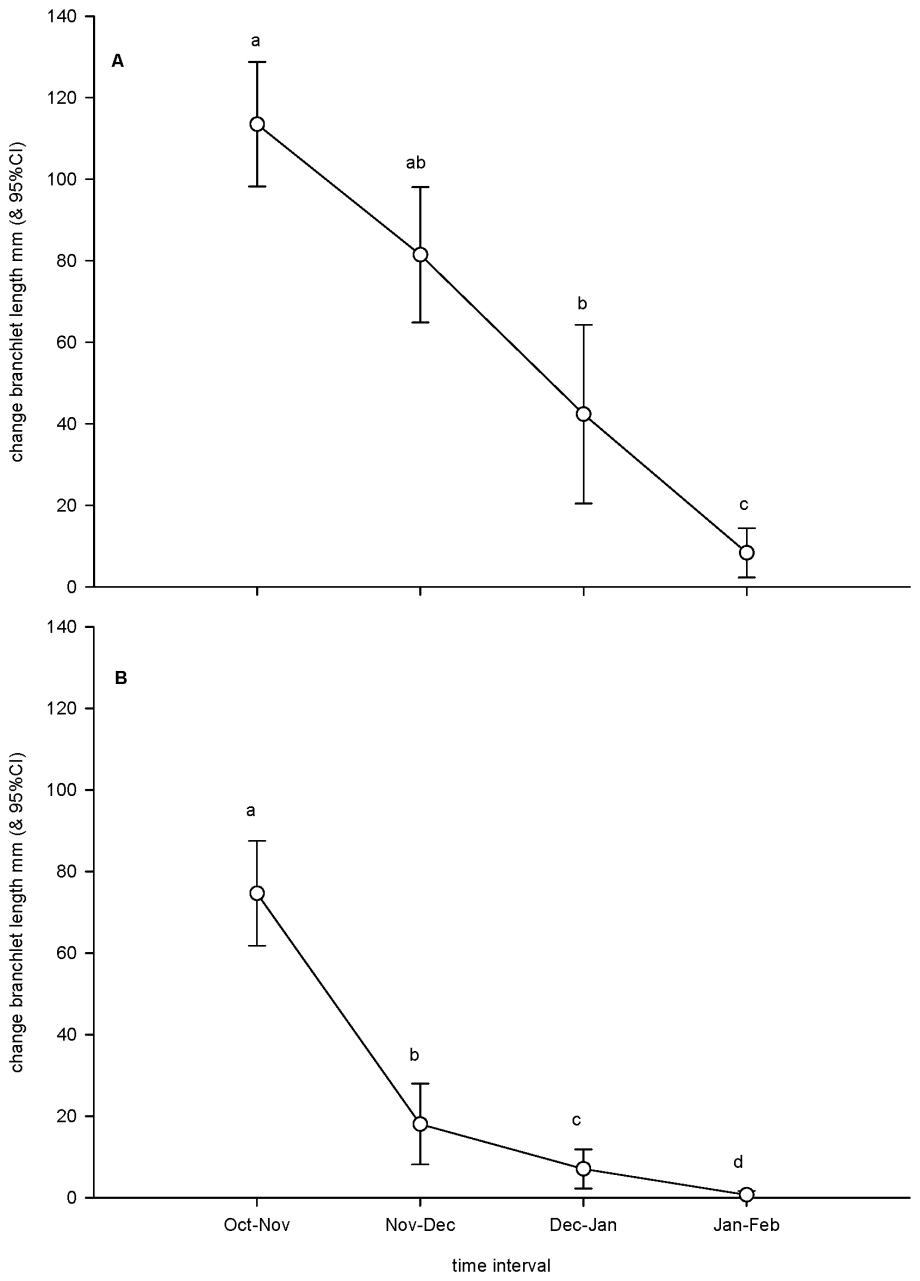
Vigour of eucalypt hosts of psyllid nymphs. **A**. Rate of extension of *E. globulus* branchlets. **B**. Rate of extension of *E. kitsoniana* branchlets. Similarities of means, determined by *post-hoc* tests, indicated by superscripted letters above bars in individual figures.

 The change in the length of *E. globulus* branchlets differed significantly according to survey interval (*F*_3, 57_ = 42.34, *P* < 0.001; Figure 2a) and declined almost linearly over time, hence, the Interval × Tree interaction term for the repeated-measures ANOVA was not significant (*F*_18, 57_ = 0.68, *P* = 0.819). The change in the length of *E. kitsoniana* branchlets also differed significantly according to survey interval (*F*_1.782, 60_ = 135.55, *P* < 0.001, probability given is after Greenhouse-Geisser adjustment; Figure 2b), however, the decline in extension was not linear as demonstrated by a statistically significant result for the interaction term (*F*_18, 60_ = 3.20, *P* < 0.001). Strong winds at Hoddle Range were responsible for the loss of many buds. For example, by the December survey only nine out of 28 original buds (32%) remained on tagged branchlets.

 The water content of *E. globulus* leaves differed significantly according to the tree and month when they were harvested and according to their age (Table 2). Not surprisingly, younger leaves had higher water contents than older leaves. Leaves from the previous growing season had the lowest water content of any leaves (Table 3). Similar findings were obtained for *E. kitsoniana* (Tables 2, 3). As expected, the water contents of both species’ leaves were negatively correlated with their specific leaf weights, i.e. an indicator of leaf toughness (Pearson correlation = -0.854 and -0.942, *P* < 0.001, respectively).

**Table 2:** Analyses of nutritional quality of host leaves.

	Source	d.f.	*F*-ratio	*P*
*E. globulus*				
Leaf water content				
Leaf pairs -2 to 13; Oct, Nov & Dec	Month	2	2.84	0.066^x^
	Tree (Month)	18	3.32	< 0.001^x^
	Branchlet (Month, Tree)	53	1.16	0.276
	Leaf pair	7	26.16	< 0.001
	Error	74		
SPAD measurements				
Leaf pairs -2 to 5; Oct to Feb	Month^†^	2.191	29.19	< 0.001
	Month × Leaf pair	12	20.14	< 0.001
	Error	284		
Leaf pairs 5 & 7; Nov to Feb	Month^†^	1.666	28.46	< 0.001
	Month × Leaf pair	3	3.11	0.030
	Error	93		
Leaf pairs 7 & 9; Dec to Feb	Month^†^	1.284	76.23	< 0.001
	Month × Leaf pair	2	1.34	0.274
	Error	44		
*E. kitsoniana*				
Leaf water content				
Leaf pairs -2 to 3; Oct	Tree	6	3.16	0.021^x^
	Branchlet (Tree)	21	0.87	0.629
	Leaf pair	2	84.46	< 0.001
	Error	37		
SPAD measurements				
Leaf pairs -2 to 3; Oct to Feb	Month^†^	2.598	112.11	< 0.001
	Month × Leaf pair	8	38.09	< 0.001
	Error	200		

Leaf water content analysed using ANOVA and SPAD measurements (chlorophyll index) analysed using repeated-measures ANOVA. Means and *post-hoc* test results are given in Table 3.

x not an exact *F*-test

† probability given is after Greenhouse-Geisser adjustment

**Table 3:** Summary of nutritional quality of host leaves.

	Leaf pair -2	Leaf pair 1	Leaf pair 3	Leaf pair 5	Leaf pair 7	Leaf pair 9	Leaf pair 11
*E. globulus*							
Oct	54.0^c^ (1.0)	61.1^b^ (2.3)	65.2^a^ (1.5)	65.2^ab^ (3.5)			
	49.7^B^	42.5^A^	43.3^A^	40.0^A^			
Nov				67.7^a^ (1.3)	68.7^a^ (1.8)	69.3^a^ (7.8)	
				42.2^A^	38.7^A^		
Dec				62.1^b^ (8.6)	64.6^b^ (1.5)	67.7^ab^ (1.3)	69.7^a^ (3.5)
					41.5^A^	37.1^A^	
*E. kitsoniana*							
Oct	52.2^c^ (2.9)	67.5^b^ (1.9)	71.7^a^ (0.8)				
	44.4^B^	38.1^A^	36.9^A^				

For each month, leaf water contents (%) are along first row and SPAD measurements (month to February 2012) are along second row. Data are means (leaf water contents; with 95% confidence intervals given in parentheses) or estimated marginal means (SPAD measurements). Similarities of means along a row (from *post-hoc* tests) indicated by superscripted letters.

 Chlorophyll index measurements for both species differed significantly according to month and leaf age (Table 2). Measurements for younger leaves were lower (less green) than those for older leaves (greener). Leaves from the previous growing season typically had the highest measurements (Table 3). Leaves of *E. globulus* required approximately one month to attain the same chlorophyll index as leaves expanded at the start of the growing season. By contrast, the leaves of *E. kitsoniana* required up to two months to attain the same chlorophyll index measurement as older leaves. Young *E. kitsoniana* leaves had a distinct yellowish colour.

### AA Availability

 Multivariate abundance analysis of AA concentrations in *E. globulus* leaves harvested from positions -2 to 5 in October and December revealed significant differences due to leaf age (*df* = 3, Likelihood Ratio (LR) = 203.0, *P* < 0.001), month (*df* = 1, LR = 361.2, *P* < 0.001) and the interaction between the two (*df* = 3, LR = 310.2, *P* < 0.001). Univariate test results indicated that concentrations of three essential AAs (Met, Thr and Val), eight non-essential AAs (Ala, Gly, Arg, Asp, Asn, Glx, Ser and Pro) and one amine metabolite (hydroxy-Pro) differed significantly according to Leaf pair and/or Month and/or the interaction between the two (Table 4). Multivariate abundance analysis of AA concentrations in *E. kitsoniana* leaves harvested from positions -2 to 3 in November and December revealed significant differences due to leaf age (*df* = 2, LR = 177.9, *P* < 0.001), month (*df* = 1, LR = 133.3, *P* < 0.001) and the interaction between the two (*df* = 3, LR = 92.3, *P* < 0.001). Univariate test results indicated that concentrations of two essential AAs (Met and Thr), four non-essential AAs (Gly, Arg, Glx and Asn) and three amine metabolites (hydroxy-Pro, Orn and GABA) differed significantly according to Leaf pair and/or Month and/or the interaction between the two factors (Table 4).

**Table 4:** Analyses of concentrations of essential and non-essential AAs and amine group metabolites.

Essential			Non			Amine group		
	Source	*P*		Source	*P*		Source	*P*
*E. globulus*								
Met	LP	0.001	Ala	M	0.023	hydroxy-Pro	M	0.001
	M	0.001	Gly	M	0.001			
	LP × M	0.001	Arg	LP	0.010			
Thr	LP	0.019		M	0.003			
	M	0.001		LP × M	0.001			
Val	LP	0.003	Asp	M	0.003			
	M	0.043	Asn	LP	0.001			
	LP × M	0.001		M	0.005			
				LP × M	0.001			
			Glx	M	0.001			
			Ser	LP	0.003			
				M	0.001			
			Pro	M	0.001			
				LP × M	0.024			
*E. kitsoniana*								
Met	LP	0.001	Gly	M	0.001	hydroxy-Pro	M	0.004
	M	0.004	Arg	LP	0.014	Orn	LP	0.011
	LP × M	0.006	Glx	M	0.001	GABA	M	0.012
Thr	LP	0.011		LP × M	0.004			
			Asn	LP	0.001			

Probabilities given are statistically significant univariate results arising from multivariate abundance analysis of amino acid concentrations. LP = leaf pair and M = month. Concentrations of all AAs and amine group metabolites in both eucalypts in both months are given in Tables S1 to S4 in File S1.

### Psyllid-Host Interactions

**Figure 3:**
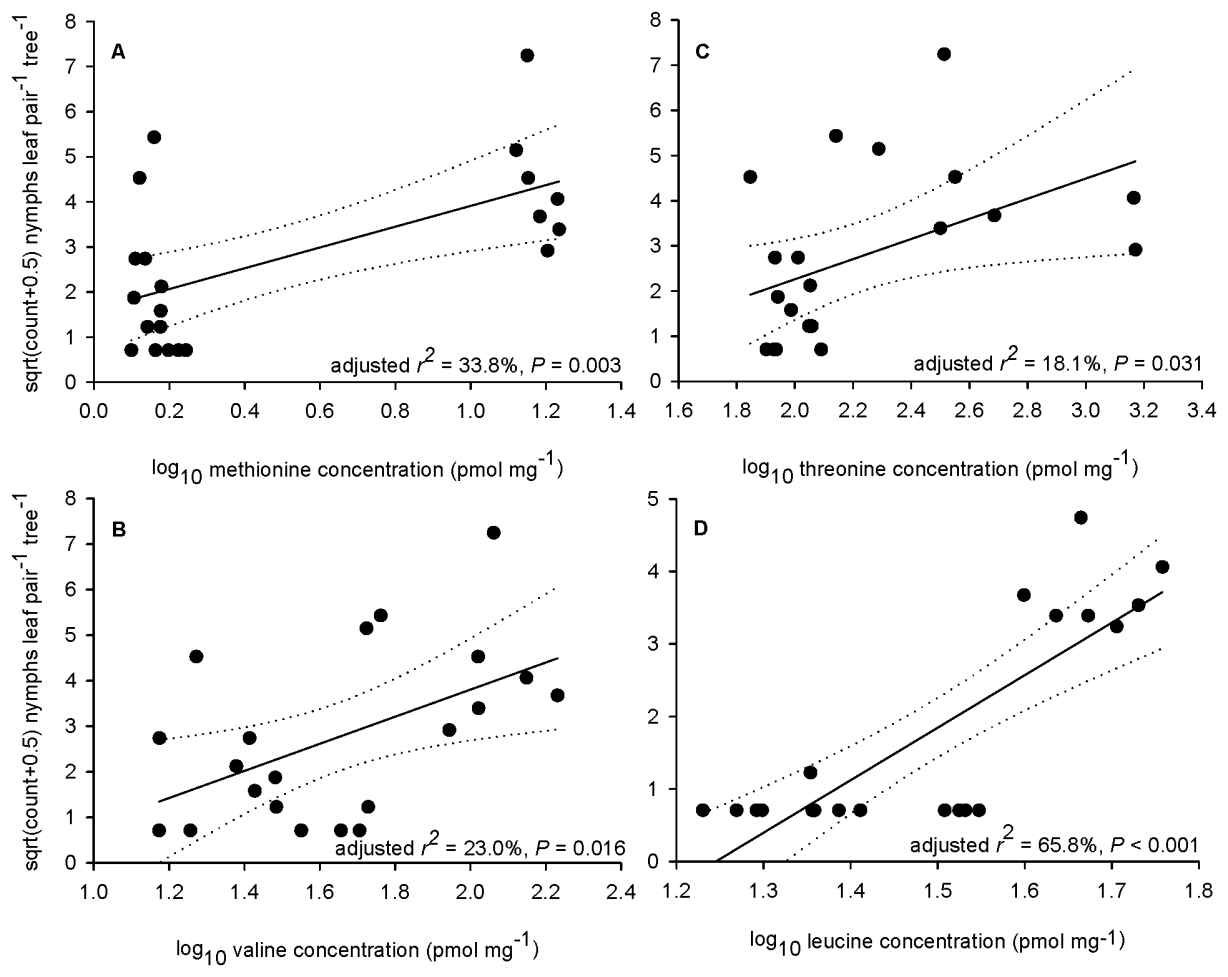
Abundance of psyllid nymphs *versus* foliar amino acid concentrations. **A**. Methionine and *C. eucalypti*. **B**. Valine and *C. eucalypti*. **C**. Threonine and *C. eucalypti*. **D**. Leucine and *C. bipartita*. Nymphal counts are for the October survey. Figures show regression lines and associated 95% confidence intervals.

 There were no statistically significant correlations between the abundances of *C. eucalypti* nymphs in buds and branchlet length, leaf water content (youngest leaf) or chlorophyll index measurement (youngest leaf) in either October or December (results not presented). Factor analysis of AA data for October created three factors that accounted for just over 75% of the variability in the data, i.e. PC1 = 44.8%, PC2 = 21.8% and PC3 = 8.5%. The same analysis of December AA data created four factors that accounted for similar variability in the data, i.e. PC1 = 31.5%, PC2 = 25.9%, PC3 = 10.5% and PC4 = 7.3%. Stepwise regressions identified the same six essential AAs (Ile, Leu, Thr, Val, Lys and Met) as statistically significant in explaining the abundance of *C. eucalypti* nymphs in both October and December (Table 5). Raw data graphing of October data revealed that, of the essential AAs, Met, Val and Thr exhibited statistically significant positive relationships with the abundance of *C. eucalypti* nymphs (Figures 3a-c). Lys exhibited a statistically significant negative relationship (adjusted *r* ^2^ = 31.4%, *P* = 0.005) with nymphal abundance (regression not shown). Graphing of December data suggested that Val and Thr exhibited statistically significant positive relationships with the abundance of *C. eucalypti* nymphs but removal of a single high nymphal count rendered these two relationships statistically non-significant.

**Table 5:** Models of abundance of psyllid nymphs *versus* foliar amino acids.

	adj. *r* ^2^	d.f.	*F*-ratio	*P*
*C. eucalypti*				
Oct				
Ile, Leu, Thr & Val + 8 non-essential AAs [PC1]	18.6%	1	5.58	0.029
Lys(-ve) & Met + 2 non-essential AAs + 2 amine metabolites [PC2]	38.3%	2	7.21	0.005
1 non-essential AA + 1 amine metabolite [PC3]	43.8%	3	6.19	0.005
Dec				
Ile, Leu, Thr & Val + 5 non-essential AAs [PC1]	10.8%	1	5.83	0.021
Lys(-ve) & Met + 3 non-essential AAs + 2 amine metabolites [PC2]	34.7%	2	11.64	< 0.001
1 non-essential AA + 1 amine metabolite [PC3]	33.0%	3	7.56	< 0.001
Trp [PC4]	47.0%	4	9.86	< 0.001
*C. bipartita*				
Oct				
Ile, Leu, Thr & Val + 4 non-essential AAs [PC1]	62.2%	1	32.27	< 0.001
Lys, Met(-ve) & Trp + 4 non-essential AAs + 2 amine metabolites [PC2]	69.4%	2	22.60	< 0.001
Met + 1 non-essential AA + 1 amine metabolite [PC3]	67.9%	3	14.41	< 0.001

Models are stepwise regressions using groupings of amino acids based on their component factor scores.

 As with *C. eucalypti*, there were no statistically significant correlations between the abundances of *C. bipartita* nymphs in buds and branchlet length, leaf water content (youngest leaf) or chlorophyll index measurement (youngest leaf) in October (results not presented). As a consequence of wind damage of tagged branchlets, there were insufficient nymphal abundance data for December to calculate a correlation matrix. Factor analysis of AA data for November created three factors that accounted for almost 76% of the variability in the data, i.e. PC1 = 32.0%, PC2 = 29.1% and PC3 = 14.8%. Stepwise regressions identified seven essential AAs (Ile, Leu, Thr, Val, Lys, Met and Trp) as statistically significant in explaining the abundance of *C. bipartita* nymphs in October (Table 5). Raw data graphing revealed statistically significant positive relationships for five of these AAs (Ile, Leu, Thr, Val and Met) and one statistically significant negative relationship (Lys). However, because nymphs were predominantly confined to buds, most of these relationships were greatly influenced by the pronounced clustering of zero and positive nymphal counts. In some instances, counts of no nymphs overlapped with positive counts at the same AA concentration. Of the six statistically significant relationships, only that for Leu did not exhibit both these traits (Figure 3d).

## Discussion

 My findings support the hypothesis that shoot feeding by psylloids could be a nutritionally beneficial adaptive strategy since water and a number of amino acids are more concentrated in younger compared to older leaves. However, leaf water content was not correlated with the abundance of either psyllid. Another surprising finding was that host vigour (branchlet length) was not related to the abundance of nymphs. Chlorophyll index measurements were also not related to the abundance of nymphs. While the variables I measured to quantify leaf quality are continuous, the occurrence of nymphs was often not continuous (in particular of *C. bipartita* nymphs) which undermines the use of regression. Therefore, to quantify how specific amino acids influence nymphal abundance will at some future time require the use of artificial diets. The findings of this field study reveal that certain amino acids exhibit significant variations in concentration according to leaf age, time of year and sometimes the interaction between the two; the influence of variations in these amino acids are considered the most promising to investigate in relation to the abundance of each psyllid species. In the case of *C. eucalypti*, the higher concentrations of three essential amino acids, namely methionine, valine and threonine, in younger leaves may explain greater abundances of nymphs. The negative relationship between the concentration of lysine and the abundance of *E. eucalypti* nymphs reflects the fact that this amino acid occurs in higher concentration in older leaves. Since nymphs do not hatch near old leaves and do not disperse far from protective microhabitats, this relationship is considered unlikely to be biologically relevant. Higher concentrations of certain amino acids (such as lysine) could conceivably act to deter adult feeding on older leaves but such concentrations would not be experienced in locations preferred for oviposition. Interestingly, the lab study revealed that *C. bipartita* is the more fecund of the two species when reared on hosts grown under identical conditions (and in the absence of strong winds). In the case of *C. bipartita*, higher concentrations of methionine, valine, threonine, isoleucine and leucine in younger leaves may explain greater abundances of nymphs. 

 To study hemipteran nutritional ecology, Douglas [43] advocated the use of EDTA-facilitated exudation of phloem sap. However, this technique requires that the petioles of excised leaves should be immersed immediately in the solution before the pores in the sieve plates of phloem elements are sealed by calcium ions. Using comparatively large aphids, Sandström [10] advocated for the use of laser stylectomy as the most reliable method for gathering phloem sap but worked with potted plants which were taken into the lab when sap collection was to occur and admitted that the procedure was “very time-consuming”. Interestingly, Riens et al. [44], using stylectomy, found that phloem sap concentrations of amino acids were not substantially different from cytosolic (intracellular fluid) concentrations. Clearly neither EDTA-facilitated exudation nor stylectomy can be used in the field, especially if many leaves need to be harvested (which is essential to acquire results representative of between and within-plant variation in nutritional quality). Moreover, Turgeon and Wolf [45] noted that neither EDTA-facilitated exudation nor stylectomy “provides a complete and artefact-free picture of the contents of moving phloem sap”. Given that the study of psyllid nutritional ecology is at such an early stage, I consider quantitation of amino acids in whole leaves essential for the identification of patterns linking psyllid distribution and abundance to host quality. In further support of my approach, it is reassuring that Merchant et al. [46] identified four essential (Met, Phe, Thr and Val), seven non-essential (Ala, Gly, Gln, Asx, Glx, Ser and Pro) and “other” amino acids as well as GABA in phloem sap bled from the stems of 12 month old *E. globulus*. These authors reported that glutamine was the most abundant amino acid (approx. 40% of total amino-N in control plants) followed roughly equally by alanine, glycine, glutamic acid and phenylalanine (each approx. 7 to 10% of total amino-N). Using whole *E. globulus* leaves of different ages, Gln, Ala, Gly, Glx and Phe were found to represent between 5.1-12.9%, 6.9-14.8%, 1.7-2.8%, 26.7-38.9% and 1.7-5.3% (October 2011) and 2.3-3.3%, 13.4-14.4%, 2.3-2.9%, 30.1-35.9% and 7.1-7.7% (December 2011), respectively, of the total amino-N recorded during this study. These percentages correspond closely to those presented in Merchant et al. [46] providing support for the quantitation of amino acid concentrations in whole leaves as a surrogate for phloem sap concentrations.

 The premise that the study of phloem-feeding insect nutrition must involve quantitation of nutrients in host sap may not be as fundamental to understanding psyllid ecology as it is to aphids. For example, histological studies of the stylet tracks of four lerp-forming species by Woodburn and Lewis [19] found that they draw nutrients from a range of plant tissues. Three species (*Cardiaspina albitextura*, *Creiis costatus* and *Lasiopslla rotundipennis*) were found to feed from the sheathing parenchyma and phloem of small vascular bundles while the fourth species (a *Glycaspis* species) was found to feed from phloem cells of large vascular bundles. Feeding by nymphs of *Cardiaspina* species causes localised leaf chlorosis; Woodburn and Lewis considered that the tissue damage associated with this feeding resembled that of naturally senescing leaves and suggested that this species was able to augment phloem nutrients with those from palisade mesophyll. In another histological study, which also included a *Glycaspis* species (namely *G. brimblecomei*) as well as two *Ctenarytaina* species (including *C. eucalypti*), psyllid stylet tracks were only found to end in vascular tissues in about 50% of instances [47]. These authors also reported that the survival of *C. eucalypti* nymphs did not differ with the leaf type (juvenile or adult) or treatment (de-waxed *versus* an intact wax layer) they were given. Most interestingly, using electrophysiology, Ullman and McLean [48] reported that pear psylla (*Psylla pyricola*) fed on all leaf cell types but that ingestion from xylem, bundle sheath cells and phloem was more common than ingestion from non-vascular tissues. Lastly, Sharma et al. [21] reported that first and second instar nymphs of a *Glycaspis* species feed on upper-mesophyll but not phloem. Evidently, different species of psyllid and even stadia feed from different tissues and the time spent feeding from phloem may be highly variable.

 The immature stages of all psyllids are highly susceptible to desiccation and the taxon as a whole exhibits a range of adaptations linked to the acquisition and retention of moisture [49,50]. Female *Ctenarytaina* posses elongate *Terminalia* which they use to insert their eggs into tight crevices such as are produced by the opposite, sessile leaf pairs which enclose the apical buds of their hosts. This bud architecture maintains a high humidity microclimate around the meristem which, possibly in combination with the waxy filaments produced by the nymphs, protects nymphs from desiccation. Large numbers of *C. bipartita* nymphs can even induce the young leaves of host buds to form leaf rolls which are usually full of condensation [33]. A similar phenomenon has been reported for another Australian psyllid, namely *Trioza eugeniae* [51]. The disappearance of nymphs after December was unexpected. *C. eucalypti* were not absent from the trees after December but were less abundant and occurred on branchlets higher in the canopy. *C. bipartita* appeared to have been absent from the trees, presumably because most of their canopies were either in intermediate or adult foliage for which adults are not host specific [47,52,53]. The very slow rate of branchlet extension by mid to late summer was associated with a decrease in the size of apical buds and it is probable that adults could no longer find sites suitable for oviposition (or perhaps were deterred by the shading of shoots by branches higher in the canopy).

 An ancestral requirement for protection against desiccation may have been the selective pressure behind the evolution of lerp formation since these structures also maintain a humid microclimate beneath them [54]. A beneficial consequence of oviposition in buds is that nymphs feed where the host is directing nutrients to support vegetative development – but was nutrition the selective pressure that drove the evolution of this behaviour? The findings of this study cannot shed any light on this question but the likelihood that nymphal nutrition has been the primary influence on oviposition behaviour seems limited given that many psyllids harbour nutritional symbionts. For example, in *C. eucalypti*, *Carsonella* and secondary symbionts exhibit a high degree of complementarity in their ability to supply psyllids with certain amino acids [55] (but note that bacterial symbionts are not capable of sterol synthesis [56]). Interestingly, three species of *Pachypsylla* and *Cecidotrioza sozanica* which either cause the formation of galls or use galls induced by other psyllid species lack secondary symbionts [57]. These authors suggested that the supply of a nutritionally balanced diet by gall tissues may have favoured the loss or disfavoured the acquisition of nutritional symbionts. If the composition of eucalypt-feeding psylloid diets is as replete in essential and non-essential amino acids as the data of Merchant et al. [46] and this study suggest, one might wonder what contribution nutritional symbionts provide their hosts. Perhaps the functional significance of nutritional symbionts is only operant when the psyllid’s eucalypt hosts experience drought or flooding. The findings presented in [46] reveal that stressors such as these markedly alter the composition of eucalypt phloem sap. If it is when eucalypt physiology is abnormal that symbionts become essential to psyllid survival, this may provide a more holistic (tree-psyllid-symbiont) explanation of their outbreaks than was originally able to be presented by White [31]. Given the importance of Australian psyllids to eucalypts overseas [58], and their functional significance in natural forest ecosystems, it is vital that we understand their nutritional ecology in greater depth. In addition, although a systematic perspective suggests that the nutritional ecology of aphids provides the most obvious parallel to use to compare with that of psyllids, the few published works relating to psyllid nutrition suggests that there may be some fundamental differences between these two groups. It could be that complete reliance on phloem-feeding may be a more derived trait in aphids than in psyllids.

## Supporting Information

File S1 **Combined file containing supporting tables.**
(DOCX)
