# Supplementary material for: Shoot Feeding as a Nutrient Acquisition Strategy in Free-Living Psylloids
Source: PLoS One. 2013 Oct 23;8(10):e77990. doi: 10.1371/journal.pone.0077990 (PMC3806811; doi:10.1371/journal.pone.0077990)
Supplement: File S1 — Combined file containing supporting tables. (DOCX) [file pone.0077990.s001.docx]

**Table S1.** Concentrations (mean ± SE; p mol mg^-1^) of essential and non-esssential AAs and amine group metabolites in leaves of *E. globulus* in October 2011. Leaf pair -2 = oldest leaves (last growing season) and leaf pair 5 = youngest leaves (current growing season).

|  | Leaf pair -2 | Leaf pair 1 | Leaf pair 3 | Leaf pair 5 | Leaf pair 7 | Leaf pair 9 |
| --- | --- | --- | --- | --- | --- | --- |
|  | *n* = 7 | *n* = 7 | *n* = 7 | *n* = 7 | (not expanded) | (not expanded) |
| *Essential* |  |  |  |  |  |  |
| Ile | 31.3 ± 4.5 | 20.9 ± 2.1 | 15.9 ± 1.7 | 49.4 ± 7.1 |  |  |
| Leu | 32.6 ± 5.1 | 25.1 ± 4.9 | 23.3 ± 3.3 | 52.5 ± 6.8 |  |  |
| Lys | 78.7 ± 21.1 | 33.6 ± 4.3 | 11.2 ± 0.6 | 17.5 ± 2.5 |  |  |
| Met | 0.45 ± 0.06 | 0.45 ± 0.06 | 16.8 ± 2.4 | 14.3 ± 0.6 |  |  |
| Phe | 262.2 ± 22.8 | 259.5 ± 27.9 | 160.7 ± 18.7 | 199.0 ± 29.9 |  |  |
| Thr | 96.9 ± 6.7 | 98.0 ± 8.4 | 113.0 ± 23.1 | 659.5 ± 212.2 |  |  |
| Trp | 40.7 ± 9.6 | 16.5 ± 3.6 | 14.6 ± 2.7 | 36.3 ± 17.4 |  |  |
| Val | 35.6 ± 5.3 | 26.2 ± 5.5 | 36.4 ± 7.6 | 110.0 ± 14.1 |  |  |
| *Non-essential* |  |  |  |  |  |  |
| Ala | 375.6 ± 80.7 | 750.7 ± 100.9 | 852.3 ± 127.4 | 978.1 ± 100.1 |  |  |
| Gly | 119.6 ± 13.3 | 81.6 ± 5.8 | 95.4 ± 12.3 | 366.3 ± 69.2 |  |  |
| Arg | 10.5 ± 5.4 | 4.5 ± 1.8 | 43.5 ± 11.5 | 437.4 ± 101.0 |  |  |
| Tyr | 27.0 ± 3.4 | 21.6 ± 1.4 | 11.8 ± 0.9 | 17.2 ± 2.0 |  |  |
| Asx | 1306.2 ± 187.4 | 918.5 ± 100.4 | 607.8 ± 120.2 | 1062.1 ± 145.1 |  |  |
| Glx | 2069.0 ± 208.8 | 1688.0 ± 191.1 | 1876.7 ± 260.4 | 3155.5 ± 318.2 |  |  |
| Asn | 35.8 ± 3.4 | 41.5 ± 3.2 | 40.1 ± 7.0 | 260.2 ± 85.0 |  |  |
| Gln | 288.5 ± 78.8 | 301.0 ± 43.4 | 544.9 ± 179.3 | 1723.9 ± 539.2 |  |  |
| Ser | 282.9 ± 32.3 | 359.5 ± 49.7 | 976.4 ± 233.2 | 2724.1 ± 453.4 |  |  |
| Cys | 0.31 ± 0.05 | 0.28 ± 0.04 | 3.1 ± 1.2 | 3.3 ± 0.8 |  |  |
| Pro | 140.7 ± 13.6 | 156.9 ± 15.6 | 170.2 ± 25.8 | 294.6 ± 47.9 |  |  |
| *Amine group* |  |  |  |  |  |  |
| hydroxy-Pro | 6.7 ± 2.1 | 23.0 ± 7.3 | 37.1 ± 8.3 | 26.7 ± 4.4 |  |  |
| Orn | 7.0 ± 1.3 | 4.7 ± 0.7 | 0.5 ± 0.2 | 2.3 ± 0.7 |  |  |
| GABA | 88.9 ± 13.1 | 149.7 ± 19.5 | 151.5 ± 47.0 | 173.8 ± 32.3 |  |  |

**Table S2.** Concentrations (mean ± SE; p mol mg^-1^) of essential and non-esssential AAs and amine group metabolites in leaves of *E. globulus* in December 2011. Leaf pair -2 = oldest leaves (last growing season) and leaf pair 9 = youngest leaves (current growing season).

|  | Leaf pair -2 | Leaf pair 1 | Leaf pair 3 | Leaf pair 5 | Leaf pair 7 | Leaf pair 9 |
| --- | --- | --- | --- | --- | --- | --- |
|  | *n* = 7 | *n* = 7 | *n* = 7 | *n* = 6 | *n* = 7 | *n* = 6 |
| *Essential* |  |  |  |  |  |  |
| Ile | 14.9 ± 2.7 | 12.4 ± 1.2 | 16.8 ± 2.2 | 16.2 ± 2.3 | 9.7 ± 1.8 | 12.1 ± 3.4 |
| Leu | 18.0 ± 3.0 | 15.4 ± 1.5 | 19.2 ± 2.0 | 19.8 ± 2.4 | 14.2 ± 2.2 | 17.1 ± 2.5 |
| Lys | 42.2 ± 5.2 | 39.3 ± 8.6 | 53.1 ± 8.6 | 50.6 ± 12.7 | 14.3 ± 4.1 | 7.6 ± 0.8 |
| Met | 0.27 ± 0.03 | 0.31 ± 0.04 | 0.34 ± 0.07 | 2.8 ± 2.4 | 10.7 ± 1.8 | 12.3 ± 1.5 |
| Phe | 146.6 ± 8.0 | 139.3 ± 17.9 | 228.7 ± 44.8 | 235.0 ± 26.9 | 169.4 ± 52.3 | 148.1 ± 35.7 |
| Thr | 65.4 ± 4.9 | 59.3 ± 5.5 | 83.3 ± 6.8 | 87.8 ± 13.5 | 54.8 ± 12.3 | 91.1 ± 40.0 |
| Trp | 50.7 ± 13.1 | 46.9 ± 10.6 | 66.6 ± 16.6 | 70.5 ± 10.1 | 41.5 ± 7.8 | 40.6 ± 14.4 |
| Val | 21.6 ± 3.3 | 17.9 ± 1.6 | 22.0 ± 2.4 | 18.3 ± 2.2 | 13.7 ± 2.1 | 24.0 ± 9.0 |
| *Non-essential* |  |  |  |  |  |  |
| Ala | 289.3 ± 30.3 | 273.9 ± 38.3 | 416.7 ± 68.7 | 456.8 ± 81.9 | 378.0 ± 70.8 | 346.3 ± 45.7 |
| Gly | 57.1 ± 4.4 | 57.2 ± 6.5 | 67.1 ± 8.1 | 72.0 ± 12.2 | 39.4 ± 7.6 | 51.3 ± 8.2 |
| Arg | 11.8 ± 3.6 | 4.9 ± 2.5 | 3.5 ± 2.2 | 4.0 ± 2.2 | 5.6 ± 2.3 | 7.5 ± 2.5 |
| Tyr | 23.8 ± 2.4 | 20.6 ± 1.6 | 25.9 ± 1.8 | 23.5 ± 2.0 | 14.5 ± 2.0 | 15.1 ± 3.0 |
| Asx | 166.0 ± 40.6 | 213.7 ± 61.6 | 1831.5 ± 1580.1 | 468.4 ± 173.7 | 217.5 ± 118.9 | 89.5 ± 40.1 |
| Glx | 794.3 ± 127.9 | 714.7 ± 82.2 | 996.6 ± 211.3 | 1010.0 ± 252.4 | 567.3 ± 181.0 | 440.4 ± 118.5 |
| Asn | 27.6 ± 1.3 | 25.4 ± 1.2 | 28.2 ± 1.7 | 29.1 ± 2.9 | 15.8 ± 3.9 | 20.4 ± 3.7 |
| Gln | 68.3 ± 7.4 | 61.4 ± 11.1 | 71.7 ± 11.4 | 75.7 ± 14.6 | 66.6 ± 16.8 | 95.1 ± 16.4 |
| Ser | 99.2 ± 10.3 | 104.3 ± 13.9 | 142.5 ± 16.6 | 196.2 ± 21.0 | 248.9 ± 32.5 | 526.9 ± 112.0 |
| Cys | 0.28 ± 0.05 | 0.27 ± 0.06 | 0.30 ± 0.03 | 0.72 ± 0.21 | 1.02 ± 0.11 | 0.99 ± 0.14 |
| Pro | 72.4 ± 5.8 | 69.4 ± 7.3 | 108.2 ± 11.1 | 121.7 ± 12.4 | 75.8 ± 14.7 | 98.6 ± 12.6 |
| *Amine group* |  |  |  |  |  |  |
| hydroxy-Pro | 2.0 ± 0.5 | 2.3 ± 0.6 | 3.2 ± 1.1 | 10.7 ± 4.9 | 11.4 ± 2.0 | 14.6 ± 1.2 |
| Orn | 6.9 ± 0.6 | 6.2 ± 1.0 | 7.5 ± 1.1 | 6.6 ± 1.5 | 0.90 ± 0.69 | 0.97 ± 0.45 |
| GABA | 174.4 ± 71.2 | 101.0 ± 31.2 | 139.6 ± 51.0 | 210.6 ± 69.5 | 166.2 ± 30.4 | 279.2 ± 90.3 |

**Table S3.** Concentrations (mean ± SE; p mol mg^-1^) of essential and non-esssential AAs and amine group containing metabolites in leaves of *E. kitsoniana* in November 2011. Leaf pair -2 = oldest leaves (last growing season) and leaf pair 3 = youngest leaves (current growing season).

|  | Leaf pair -2 | Leaf pair 1 | Leaf pair 3 | Leaf pair 5 |
| --- | --- | --- | --- | --- |
|  | *n* = 6 | *n* = 7 | *n* = 7 | (not expanded) |
| *Essential* |  |  |  |  |
| Ile | 23.9 ± 3.2 | 22.1 ± 3.1 | 39.5 ± 1.7 |  |
| Leu | 24.2 ± 2.9 | 24.3 ± 2.4 | 47.3 ± 2.3 |  |
| Lys | 107.6 ± 19.5 | 49.9 ± 4.0 | 42.9 ± 3.6 |  |
| Met | 2.6 ± 0.8 | 12.1 ± 0.5 | 13.3 ± 1.2 |  |
| Phe | 133.4 ± 27.2 | 284.6 ± 62.1 | 522.7 ± 89.6 |  |
| Thr | 94.0 ± 11.2 | 69.4 ± 11.8 | 172.1 ± 38.8 |  |
| Trp | 154.1 ± 47.4 | 21.9 ± 5.4 | 28.1 ± 5.0 |  |
| Val | 38.3 ± 7.5 | 30.8 ± 3.1 | 57.2 ± 3.0 |  |
| *Non-essential* |  |  |  |  |
| Ala | 247.8 ± 27.5 | 301.5 ± 43.3 | 274.5 ± 26.4 |  |
| Gly | 121.1 ± 5.0 | 95.5 ± 6.4 | 317.6 ± 58.3 |  |
| Arg | 178.5 ± 52.5 | 34.6 ± 4.6 | 48.9 ± 10.8 |  |
| Tyr | 39.6 ± 13.8 | 20.7 ± 2.8 | 37.4 ± 5.7 |  |
| Asx | 1077.3 ± 190.9 | 414.1 ± 43.7 | 402.0 ± 42.6 |  |
| Glx | 2006.1 ± 98.6 | 930.1 ± 82.8 | 878.0 ± 114.1 |  |
| Asn | 34.7 ± 1.6 | 37.1 ± 7.7 | 165.6 ± 62.2 |  |
| Gln | 369.2 ± 42.3 | 213.2 ± 16.5 | 311.3 ± 23.5 |  |
| Ser | 306.2 ± 25.7 | 466.7 ± 40.1 | 891.8 ± 160.2 |  |
| Cys | 16.9 ± 5.7 | 12.0 ± 1.2 | 10.5 ± 1.1 |  |
| Pro | 162.4 ± 14.6 | 213.4 ± 38.1 | 452.4 ± 105.0 |  |
| *Amine group* |  |  |  |  |
| hydroxy-Pro | 7.3 ± 3.7 | 14.3 ± 1.9 | 21.4 ± 3.8 |  |
| Orn | 13.0 ± 2.3 | 1.4 ± 0.5 | 3.0 ± 1.1 |  |
| GABA | 82.6 ± 15.6 | 54.1 ± 8.2 | 48.3 ± 4.1 |  |

**Table S4.** Concentrations (mean ± SE; p mol mg^-1^) of essential and non-esssential AAs and amine group containing metabolites in leaves of *E. kitsoniana* in December 2011. Leaf pair -2 = oldest leaves (last growing season) and leaf pair 5 = youngest leaves (current growing season).

|  | Leaf pair -2 | Leaf pair 1 | Leaf pair 3 | Leaf pair 5 |
| --- | --- | --- | --- | --- |
|  | *n* = 7 | *n* = 7 | *n* = 7 | *n* = 5 |
| *Essential* |  |  |  |  |
| Ile | 15.1 ± 1.5 | 18.6 ± 3.2 | 18.8 ± 3.7 | 39.2 ± 8.8 |
| Leu | 17.5 ± 1.9 | 20.0 ± 2.7 | 20.6 ± 2.5 | 40.4 ± 8.0 |
| Lys | 57.0 ± 9.6 | 59.0 ± 5.0 | 20.2 ± 2.1 | 28.6 ± 3.6 |
| Met | 2.0 ± 0.6 | 3.7 ± 1.4 | 9.9 ± 0.4 | 9.5 ± 0.3 |
| Phe | 112.1 ± 20.5 | 118.3 ± 11.9 | 425.3 ± 101.6 | 717.9 ± 166.6 |
| Thr | 86.1 ± 10.9 | 86.0 ± 8.1 | 125.9 ± 18.8 | 891.4 ± 284.6 |
| Trp | 54.9 ± 23.4 | 33.3 ± 5.2 | 14.2 ± 4.2 | 25.6 ± 6.8 |
| Val | 21.7 ± 1.4 | 29.6 ± 4.0 | 29.4 ± 4.8 | 70.0 ± 19.2 |
| *Non-essential* |  |  |  |  |
| Ala | 526.3 ± 107.8 | 616.0 ± 99.7 | 542.0 ± 128.1 | 696.1 ± 106.5 |
| Gly | 68.9 ± 6.6 | 65.3 ± 6.1 | 87.4 ± 11.4 | 392.6 ± 128.9 |
| Arg | 136.5 ± 35.3 | 93.4 ± 23.5 | 125.7 ± 74.8 | 374.1 ± 179.0 |
| Tyr | 20.8 ± 3.6 | 24.2 ± 3.7 | 16.4 ± 2.5 | 23.5 ± 3.9 |
| Asx | 880.8 ± 125.8 | 554.7 ± 86.8 | 844.8 ± 117.2 | 807.9 ± 115.1 |
| Glx | 1748.5 ± 222.0 | 1330.7 ± 98.8 | 1886.4 ± 260.2 | 1891.6 ± 167.8 |
| Asn | 42.8 ± 5.8 | 38.1 ± 2.7 | 158.0 ± 47.2 | 1321.5 ± 517.4 |
| Gln | 320.1 ± 37.0 | 408.8 ± 85.8 | 532.9 ± 111.5 | 896.9 ± 266.1 |
| Ser | 384.5 ± 125.5 | 311.1 ± 45.2 | 1003.4 ± 214.4 | 2207.4 ± 547.9 |
| Cys | 12.5 ± 6.0 | 13.7 ± 5.8 | 8.3 ± 0.8 | 7.1 ± 0.4 |
| Pro | 153.4 ± 33.6 | 154.8 ± 13.0 | 327.6 ± 57.0 | 2987.4 ± 1345.1 |
| *Amine group* |  |  |  |  |
| hydroxy-Pro | 4.0 ± 0.9 | 3.8 ± 1.4 | 9.5 ± 1.8 | 20.5 ± 4.6 |
| Orn | 11.2 ± 1.4 | 10.1 ± 1.8 | 0.6 ± 0.1 | 2.4 ± 0.6 |
| GABA | 157.2 ± 54.4 | 199.5 ± 47.4 | 98.7 ± 18.4 | 227.6 ± 49.0 |
